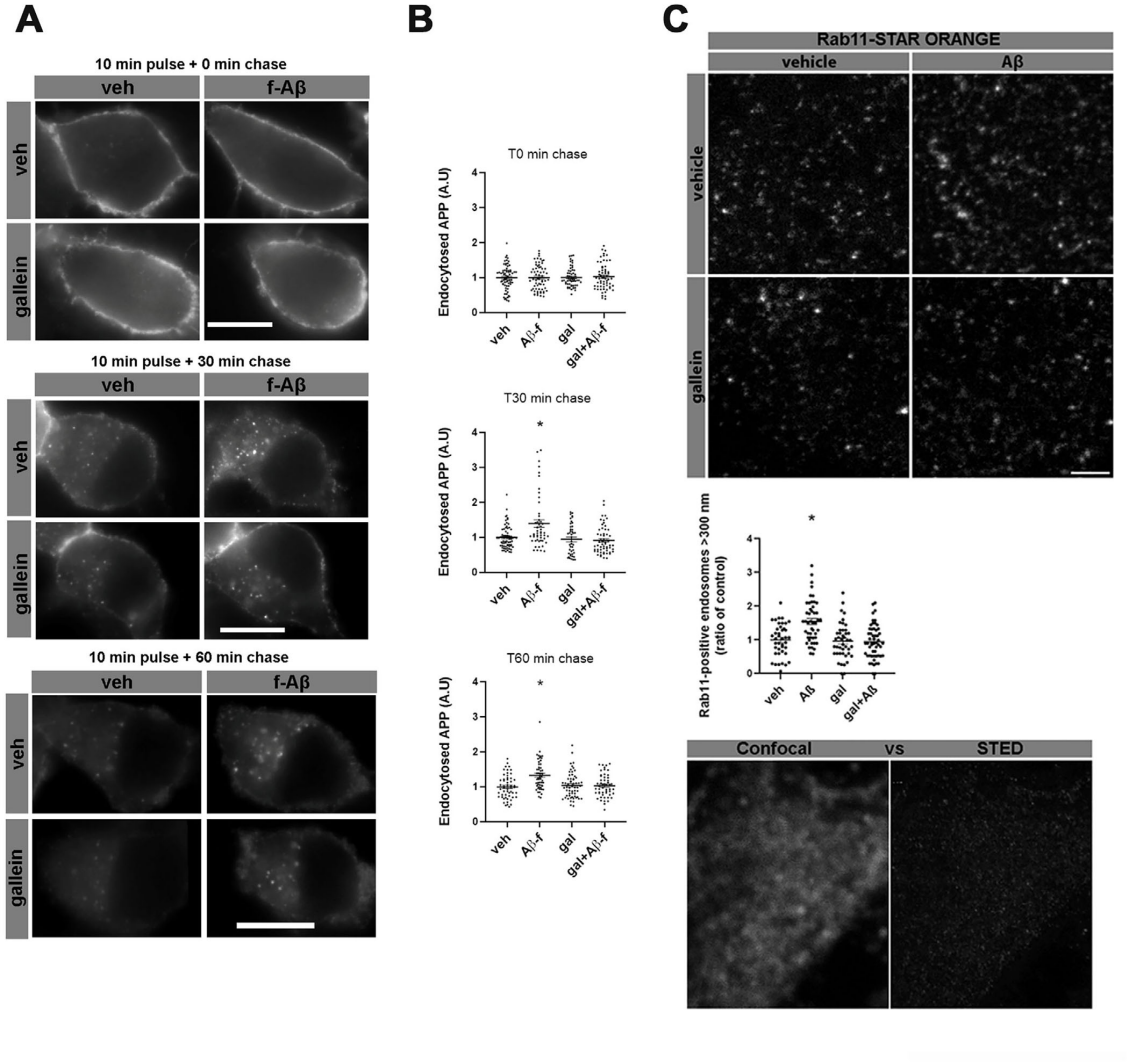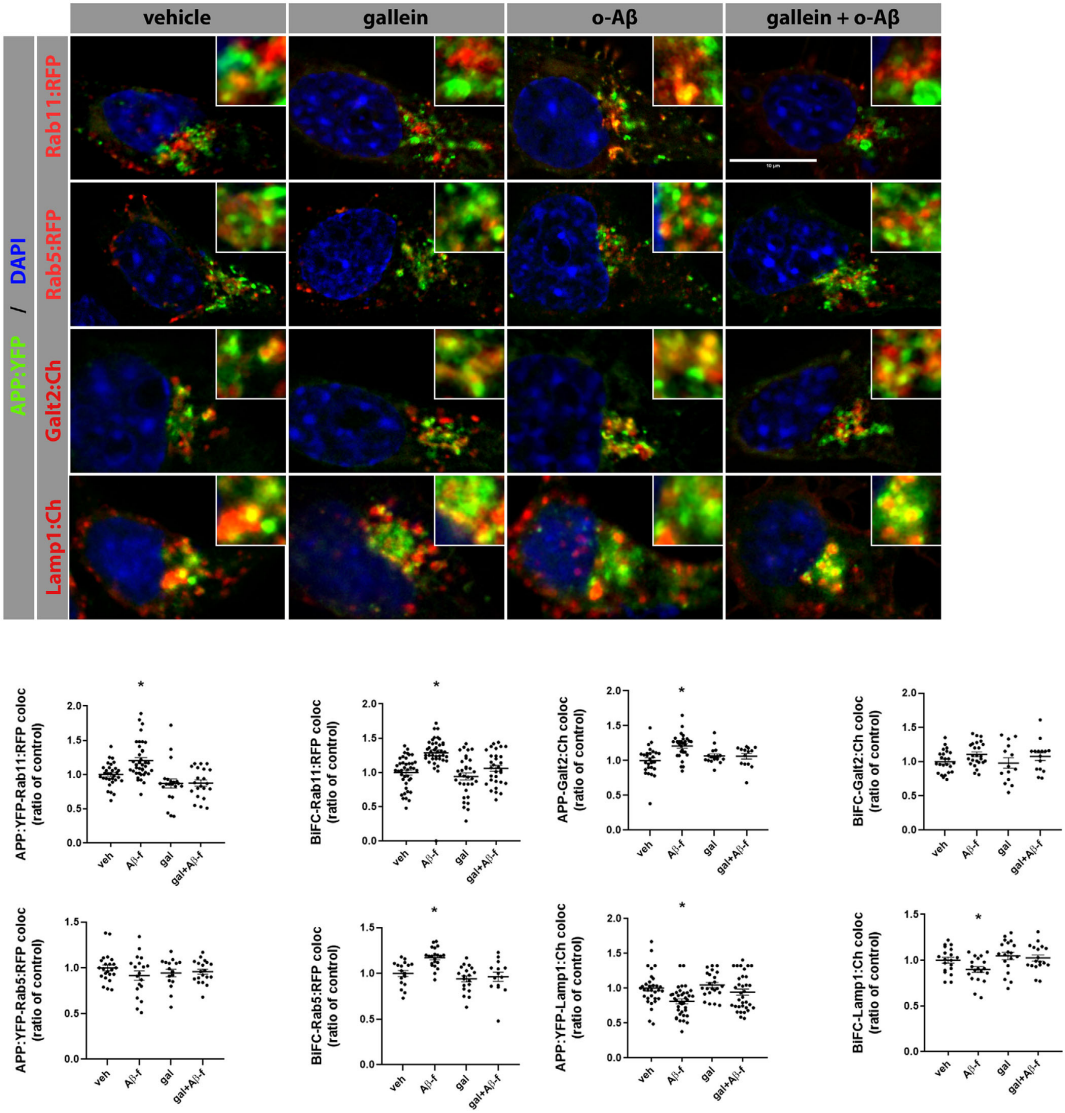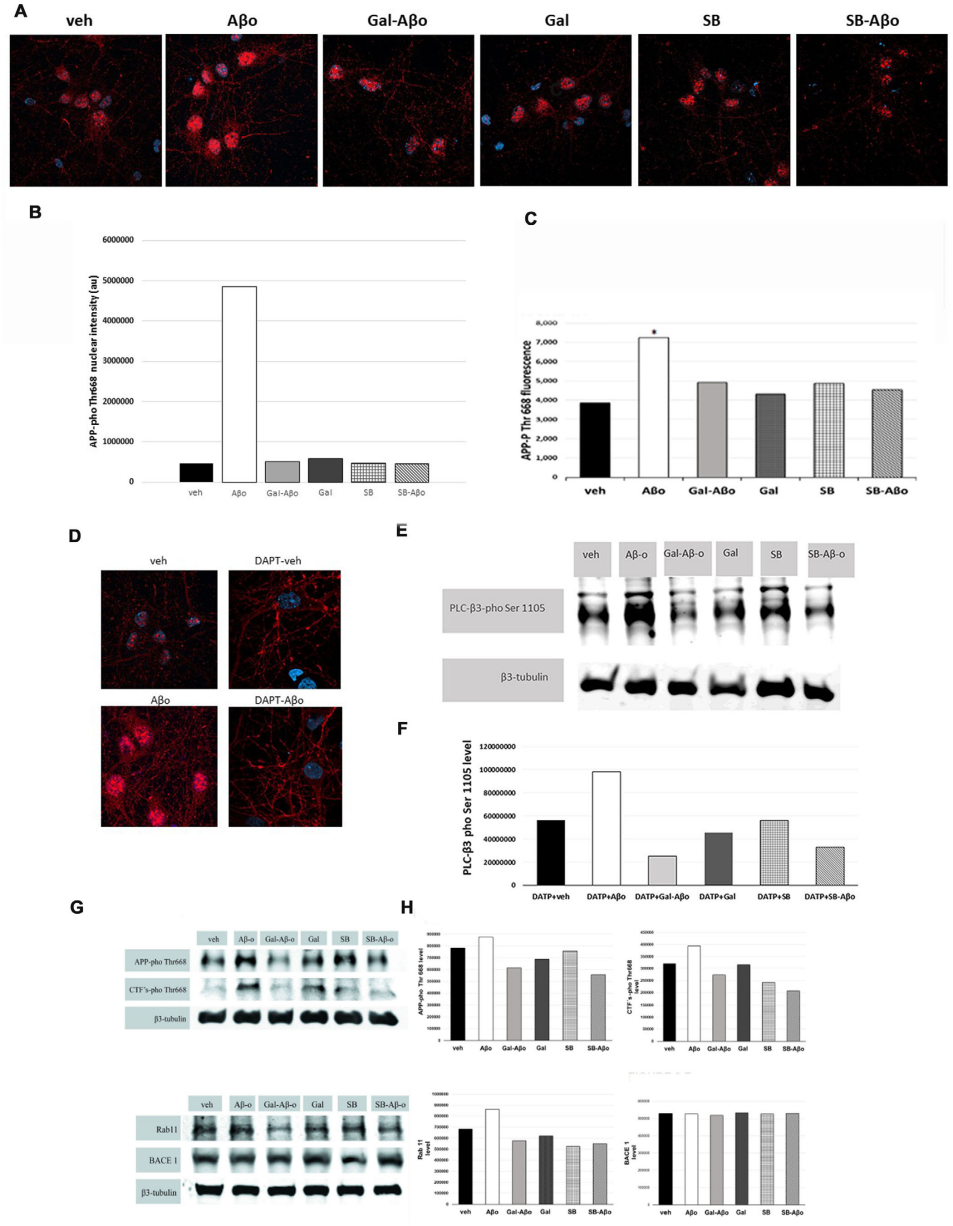# Unveiling a role of the Gβγ/PLCβ3 signaling pathway on APP endolysosomal re‐distribution and Aβ‐induced amyloidogenesis

**DOI:** 10.1002/alz.095540

**Published:** 2025-01-09

**Authors:** Romina soledad Almirón, Magdalena Antonino, Paula Marmo, Alfredo Lorenzo, Elena Anahi Bignante

**Affiliations:** ^1^ INIMEC‐CONICET‐UNC, Cordoba Argentina

## Abstract

**Background:**

Aβ accumulation is a key event driving neurotoxicity in Alzheimer’s disease. Previously, we demonstrated that oligomers of amyloid beta (oAβ) induce an increase in the levels of APP and BACE1 in Rab11‐positive endosomes, leading to the intracellular accumulation of Aβ1‐42 in human neurons derived from iPSCs (HN‐iPSCs). This vicious cycle of Aβ generation induced by Aβ itself, is pivotal for the propagation of pathology. We now aim to deeper into the pro‐amyloidogenic mechanisms underlying the enrichment of APP in Rab11‐endosomes.

**Method:**

We assessed APP distribution in N2a cells transfected with APP‐YFP/BACE1‐Ch and compartment markers fused to fluorescent tags. Cells were treated with vehicle or Gβγ inhibitor gallein (5μM), oAβ (1 µM) and 24 h later quantitative colocalization analyses were conducted. APP endocytosis rate was determined via pulse‐chase assays using 22C11 antibody. Levels of APP and AICD phosphorylated at thr668 (APP‐pho668 and AICD‐pho668) in rat cortical neurons and HN‐IPSCs were determinate by immunofluorescence. Western blotting was employed to determine the levels of APP‐pho668, βCTF‐pho668, Rab11, Rab5, and PLCβ3‐pho. Super‐resolution microscopy was utilized to quantify the number and size of Rab11‐endosomes.

**Result:**

Aβ treatment induced an enrichment of APP in Rab11‐endosomes by increasing its rate of endocytosis while decreasing its localization to lysosomes. We observed a concomitant increase in the number of large Rab11‐endosomes (greater than 300 nm). These alterations in the distribution of APP within endolysosomal compartments were mediated by Gβγ signaling, as it were prevented by pretreatment with gallein (Fig. 1, 2). Aβ treatment also provokes an increase in the levels of APP‐pho668 in process and AICD‐pho668 in nuclear speckles of cortical neurons and HN‐iPSC. Both effects were prevented with gallein or inhibitor of p38MAPK (SB) incubation. WB analysis showed an increase in APP‐pho668, βCTF‐pho668, Rab11 and PLCβ3‐pho induced by Aβ, effects mitigated by gallein and SB treatments (Fig. 3).

**Conclusion:**

These findings suggest that Aβ treatment triggers the activation of a signaling pathway involving Gβγ/PLCβ3/p38MAPK, leading to an increase in APP phosphorylation at Thr668. This event correlates with enhanced APP endocytosis and redirecting it from its normal pathway to lysosomes towards Rab11‐endosomes which facilitates its amyloidogenic processing.